# A method for generating large datasets of organ geometries for radiotherapy treatment planning studies

**DOI:** 10.2478/raon-2014-0003

**Published:** 2014-11-05

**Authors:** Nan Hu, Laura Cerviño, Paul Segars, John Lewis, Jinlu Shan, Steve Jiang, Xiaolin Zheng, Ge Wang

**Affiliations:** 1 Department of Radiation Oncology, Cancer Center,Research Institute of Surgery, Daping Hospital, Third Military Medical University, China; 2 Department of Radiation Oncology, University of California, San Diego, USA; 3 College of Bioengineering, Chongqing University, China; 4 Department of Radiology, Duke University, USA

**Keywords:** non-uniform rational B-spline technique, new geometries, statistical shape model, adaptive radiotherapy

## Abstract

**Background:**

With the rapidly increasing application of adaptive radiotherapy, large datasets of organ geometries based on the patient’s anatomy are desired to support clinical application or research work, such as image segmentation, re-planning, and organ deformation analysis. Sometimes only limited datasets are available in clinical practice. In this study, we propose a new method to generate large datasets of organ geometries to be utilized in adaptive radiotherapy.

**Methods:**

Given a training dataset of organ shapes derived from daily cone-beam CT, we align them into a common coordinate frame and select one of the training surfaces as reference surface. A statistical shape model of organs was constructed, based on the establishment of point correspondence between surfaces and non-uniform rational B-spline (NURBS) representation. A principal component analysis is performed on the sampled surface points to capture the major variation modes of each organ.

**Results:**

A set of principal components and their respective coefficients, which represent organ surface deformation, were obtained, and a statistical analysis of the coefficients was performed. New sets of statistically equivalent coefficients can be constructed and assigned to the principal components, resulting in a larger geometry dataset for the patient’s organs.

**Conclusions:**

These generated organ geometries are realistic and statistically representative.

## Introduction

In recent years, clinical linear accelerators combined with cone-beam computed tomography (CBCT) have become available, and they provide valuable 3D geometric information of patients. This combination offers the advantage of incorporating daily images into the radiotherapy process, such as setup-error correction, dose accumulation^1^–^4^, evaluation and re-planning^5^,^6^, and re-optimization^7^,^8^, which are essential for adaptive radiotherapy (ART).^9^–^15^

ART integrated with CBCT^16^, which uses the daily geometric information to adjust, in each fraction, the treatment plan to the updated patient’s anatomy and positioning, significantly improves the accuracy and success of the radiation therapy.^17^–^19^ Image registration between the planning image and daily CBCT images, dose reconstruction, and treatment evaluation were basically employed to determine whether and how the original plan needs to be adjusted. Plan re-optimization may then be applied, and a new plan is to be worked out for the new fraction of the treatment. It is an optimal compensation of uncertainties, including organ deformation, organ motion and dosimetric errors incurred in previous fractions.

In ART studies of gynecologic cancer, such as 3D organ segmentation, re-planning, and organ shape variation, it is often required the use of large medical geometrical datasets which can represent accurately all cases in a population from which the training set has been sampled. However, usually only limited datasets are available. Sometimes the size of the training dataset was considered to be the most important reason for a relatively high segmentation error.^20^ Statistical shape modeling has been proved effective for interpreting objects whose shape can vary.^21^ Individual geometric variation can be modeled based on dominating eigen modes of organ deformation.^22^ Due to the use of small training datasets, statistical shape models often constrain too much the deformation in medical image applications.^23^–^24^

The purpose of this work is to develop a novel method to generate large datasets of organ geometries from an actually acquired training dataset of limited size. A statistical shape analysis^25^–^26^ based on the principal component analysis (PCA) was used to determine the major deformation modes present in the training organ geometries. Nonuniform rational B-spline (NURBS) technique, which provides the flexibility to design a large variety of shapes, was also integrated to represent the organ surface. This approach is intended to support various tasks associated with pelvic image processing in adaptive radiotherapy by constructing statistical models of organ deformations and exploiting pelvic organ geometric morphometrics.

## Materials and methods

In this section we describe the method we propose to generate new organ geometries based on limited training datasets. First, a set of training images was acquired from daily CBCT scan. Next, surface registration and closest point searching approaches were applied to the training datasets to get the surface points correspondence between different CBCTs. In the following step, a statistical shape analysis to represent the major deformation modes of pelvic organs based on NURBS and PCA was performed; a set of coefficients corresponding to each principal component was obtained. Expectation maximization (EM) algorithm was then used to approximate and estimate the probability density function (PDF) of these coefficients. Finally, by assigning different coefficients from the PDF to the respective principal components, new realistic geometries of the organs can be obtained. A detailed description of each of these steps follows.

### Training patient dataset collection and preprocessing

CBCT images have been acquired during the image-guided radiation therapy of gynecologic cancer in a Varian Trilogy treatment machine with on-board imaging system (Varian Medical Systems, Inc., U.S.A.). The on-board imaging system, which consists of a kV x-ray source and a flat panel detector, was installed onto the gantry along an axis orthogonal to the mega voltage beam. The acquired images had 512´512 pixels, with pixel size ranging from 0.5859 to 0.625 mm and slice thickness 2.5 mm. Each CBCT volume consisted of 54 slices.

After the CBCT reconstruction, all the daily images were transferred into Eclipse treatment planning system (TPS) and co-registered. Bladder, rectum, intestines and other organs of interest were extracted from daily CBCT images through manual segmentation, thresholding, and user interaction. Each organ was defined with a series of discrete transverse contours, which are represented by a list of vertices and associated with each of the transverse image slices. The representations of contours for the respective organs were then exported from the TPS and converted to a volumetric binary stack file.

### Polygon surface generation

Accurate polygon surface models of anatomical objects have a great impact in various medical applications. They can be used as the basis for computational purposes or morphometrical analysis. In our study, polygon mesh modeling was employed to construct 3D triangular models of the female pelvic organs of interest, and were later converted into NURBS form.

The 3D polygon-surface mesh based on triangles was created from the delineated contours of each organ by using the software IsoSurf (Graham Treece, University of Cambridge, UK.). This software supports extracting triangulated surfaces from blocks of data at varying resolutions, using regularized marching tetrahedra. The original input data to IsoSurf is assumed to be from a stack of parallel images, each of the same size. Here, the binary stack files obtained from the TPS in the previous step were used as the input for isosurf. The set of polygon mesh files was generated for the corresponding organs of all the training images with controllable spatial resolution.

### Mesh registration and establishment of point correspondence

A crucial part of our study lies in establishing a point correspondence for all input shapes of each organ, which will be used as the basis for the statistical shape analysis and contribute to new geometries generation. Given the set of N segmented input training surfaces of pelvic organs, for each organ, one of the surfaces was selected as reference surface and the other N-1 training surfaces were set as target surfaces.

An interactive affine registration defined by scaling and translation transformations was initially performed between the reference surface and the N-1 training surfaces, in order to align all the organ shapes into a common coordinate frame. This common coordinate frame can be used to get an appropriate initialization for establishing point correspondence of pelvic organs.

Several approaches could be used to establish mesh-based surface point correspondence. An approach that builds upon the work for surface registration^27^ was selected here to find corresponding points on reference surface and the other N-1 training organ surface. This approach was implemented by looking over all the faces, edges, and vertices of the surface meshes to find the closest point on the target surface to each point on the reference image. For this purpose, the point on the reference surface was projected on the corresponding training surface, and the intersection of the line passing through the point that is perpendicular to the surface patches close to projected point was found. The closest point in the target mesh to the intersection was considered the corresponding point. Once point correspondences are established, a topological correlation between the reference surface and target surfaces can be built for the statistical shape analysis.

### NURBS generation of the reference surface

In order to determine the organ variation modes based on NURBS representation, NURBS modeling was performed on the reference organ surfaces. Here, NURBS-surface organ models are constructed from the imported polygon mesh models with the Rhinoceros software (McNeel, Seattle, WA, USA), which supports polygon meshes and can be used to create, edit, analyze, and translate NURBS curves, surfaces, and solids.

In Rhinoceros, several contours for each organ were obtained from the input polygon mesh of the reference surface as needed, and then several superior-inferior lines were simultaneously drawn to connect all the contours. NURBS surfaces were then fitted to match the contours by means of the Rhinoceros *loft* function.

### NURBS deformation and surface matching

Depending on the displacements of the corresponding surface points, the NURBS representation of the reference surface can be deformed to match the target training surfaces. With this matching procedure, the deformed reference surface will have the same NURBS topology as before, but will have the same shape as the target surface and can, thus, be later used in place of the target surface in the statistical shape analysis.

The surface matching can be expressed as a deformation procedure of NURBS control points based on the displacements of surface points:

[1] C2=f(C1,X1→X2),

where X1 and X2 stand for the corresponding surface points on the reference and target surfaces respectively, and C1 and C2 stand for the corresponding NURBS control points of reference surface and target surfaces respectively.

In order to bring the deformed reference surface closer to the target surface, the deformation procedure was divided into several intermediate steps to avoid the local minima problem:

[2] $$\text{S}1\to \text{S}2\Rightarrow \text{S}1\to {\text{S}}_{11}\to {\text{S}}_{12}\to {\text{S}}_{13}\ldots {\text{S}}_{1\text{n}-1}\to \text{S}2,$$

where S1 and S2 stand for the reference and target surfaces respectively, and S_11_, S_12_, S_13_, ¼S_1n-1_ stand for the intermediate shapes, which are given by:

[3] $${\text{S}}_{1\text{j}}=\text{S}1+(\text{S}2-\text{S}1)*\text{j}/\text{n }(\text{j}=1,2,3\ldots \text{n}-1).$$

n is the total number of substeps, and j is the current working substep. By dividing the deformation in different substeps, the NURBS representation of the reference surface converges to the target surface.

During every intermediate step, a NURBS warping of the reference organ shape is performed point by point. After one warping step for one control point, both surfaces may coincide at the desired position but may differ at other surface parts. For this reason, the deformation of control points is iterated until a smooth convergence is achieved.

### Re-generation of surface points

Organ deformation can be interpreted as the change of organ geometry as given by its shape, and organ shape can be parameterized by the position of a set of surface points. In order to continue with the process and obtain the major deformation modes of the pelvic organs with PCA, a set of surface points is re-sampled from the corresponding NURBS surfaces with controlled precision. This process also provides an accurate approximation of the 3D organ shape with only a few digitized NURBS surface points.

### Generating new organ geometries based on the statistical shape analysis

In the statistical shape analysis of pelvic organs, we have represented shapes by means of point distribution models (PDM)^28^, which can provide a few-parameter statistical model of organ deformation. The basic idea is to compute the mean shape and to establish, from the training set, the pattern of legal variations in shape. This is done by using PCA to quantitatively determine the major deformation modes present in a training series, which, in our case, were parameterized by sets of corresponding organ surface points. PCA defines a linear transformation that de-correlates the parameter signals of the original shape population by projecting the objects into a linear shape space spanned by a complete set of orthogonal basis vectors. The axes of the shape space are oriented along directions in which the data have its highest variance.^29^ Assuming that the corresponding points are located at equivalent positions on different instances of the shape, PCA is used to define a coordinate frame aligned to the principal axes of the data.

Given the training set of size N, PCA was performed on the coordinates of corresponding NURBS surface points {t_j_=(t_xj_,t_yj_,t_zj_), j=1,…,m}, where m is the number of surface points used in the analysis. The PDM constructed from training organ shapes is defined by:

[4] $$X=\bar{X}+Pb=\bar{X}+\sum p_{q}b_{q},$$

where *X* is a 3m-element shape vector, *X̄* is the mean of the aligned organ shapes{*X_i_*,i=1..N} in the training set. *P* is a matrix containing the first *k* eigenvectors *p_q_* of the covariance matrix *D* defined as

[5] $$D=\frac{1}{N-1}\sum_{k=1}^{N}\left(X_{i}-\bar{X}\right){\left(X_{i}-\bar{X}\right)}^{T},$$

and *b_q_* is the coefficient in the linear analysis corresponding to eigenvector *p_q_*. *D* is also involved in the computation of the eigenvalues *λ_q_* and eigenvectors *p_q_*:

[6] $$D\cdot p_{q}=\lambda_{q}\cdot p_{q}$$

By assigning a new set of coefficients *b_q_* to the principal components, new geometries of the organs can be obtained. The new coefficients *b_q_* can be sampled from statistical distributions extracted from the training data. A method reported by Cootes^30^ was employed here to analyze the probable density function (PDF) of the coefficients *b_q_*. The PDF was approximated with a mixture of Gaussian distributions derived from the kernel method:

[7] $$p_{\mathrm{mix}}\left(b_{q}\right)=\sum_{l=1}^{r}w_{l}N\left(b_{q}:\mu_{l},S_{l}\right)$$

where *N* (*b_q_* : *μ_l_*, *S_l_*) is a PDF of a gaussian with mean *μ* and covariance *S*. The Expectation Maximization (EM) algorithm^31^ was used to fit such mixture to the given data and then a set of mixture-gaussian functions for the coefficients *b_q_* was obtained.

Once the distribution function of the coefficients is known, its cumulative distribution function (CDF) can be obtained. From the CDF, a series of random coefficients can be generated with Monte-Carlo sampling. Random generation of coefficients is implemented via an inversion method. If *u* is an uniform random number over the interval (0, 1), then a random number *W* from a distribution with specified CDF *F* is obtained using *W* = *F*^−1^(*u*). The new geometry of the organ was obtained from:

[8] $$X_{\mathrm{new}}=\bar{X}+\mathrm{PW}$$

where *X_new_* stands for the new generated organ shape.

## Results

**FIGURE 1.**
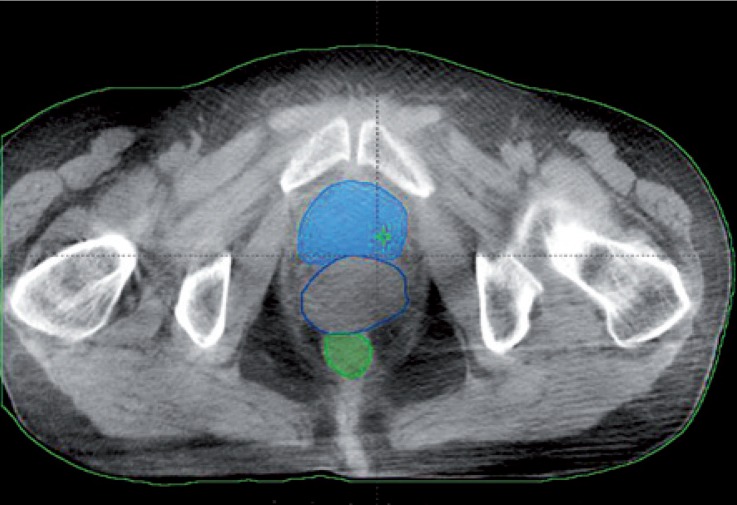
Pelvic organs segmented in cone-beam computed tomography (CBCT) images.

We acquired CBCT images from 10 patients with gynecologic cancer. 15 image sets from different days were acquired for each patient. Bladder, rectum, intestines and other organs were contoured in Eclipse TPS (Figure 1). The contours of each organ were exported from TPS for the analysis.

**FIGURE 2.**
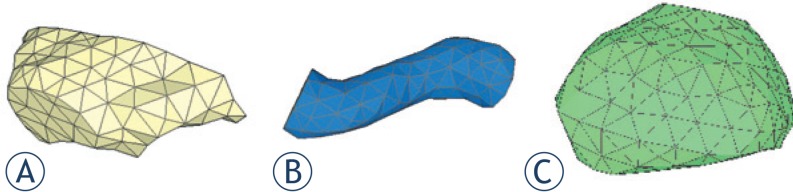
Polygon surface of pelvic organs. (A) Bladder; (B) Rectum; (C) Intestine.

With the contours of each pelvic organ, polygon surfaces were generated with the Isosurf software. Typical examples of triangular meshes for the rectal, the bladder, and the intestines are shown in Figure 2.

**FIGURE 3.**
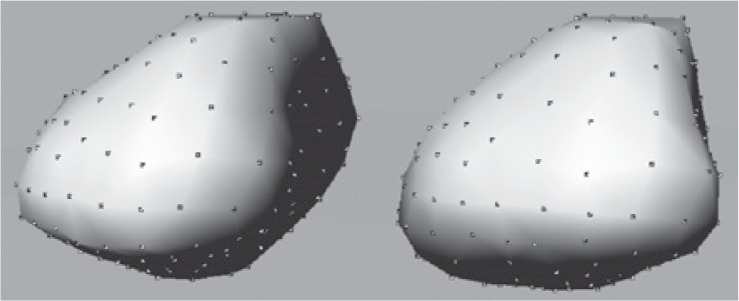
Corresponding points on two organ surface.

Point correspondence was established between the reference surface and the target training surfaces. This correspondence was achieved by a rigid transformation and a closest point search approach. Figure 3 illustrates two different surfaces of the same organ and the corresponding points.

**FIGURE 4.**
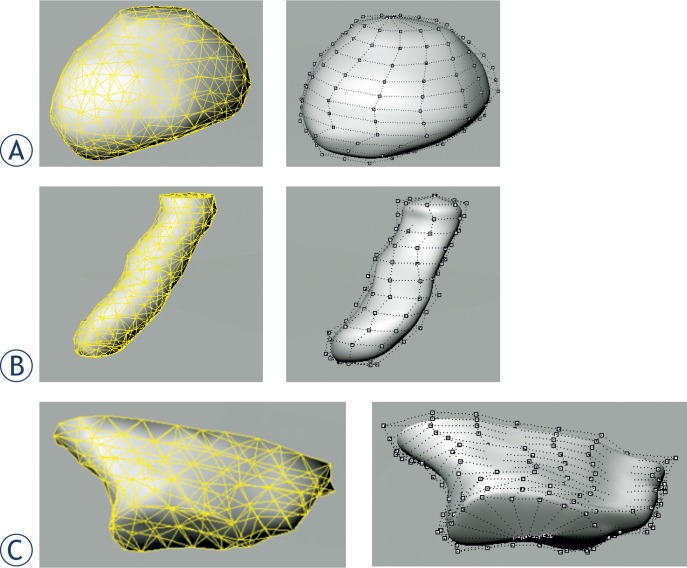
Nonuniform rational B-spline (NURBS) representation of pelvic organs converted from polygon meshes. Upper: polygon surfaces represented by triangular meshes. Lower: corresponding NURBS surfaces with control points. **(A)** Bladder; **(B)** Rectum; **(C)** Intestine.

For the reference surfaces, the polygon meshes have been converted into NURBS, which are represented by feature control points, by using the Rhinoceros software. Example NURBS representation of bladder, rectum, and intestines derived from polygon surface are shown in Figure 4. Polygon meshes (left) and NURBS control points (right) are shown.

**FIGURE 5.**
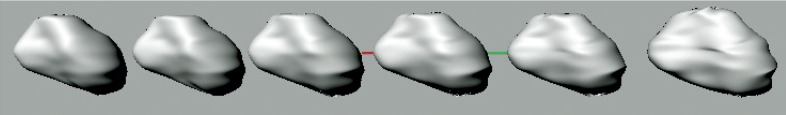
Non-uniform rational B-spline (NURBS) surface deformation with intermediate steps.

Figure 5 illustrates the deformation from the NURBS representation of a reference surface to the target surface. Intermediate steps used in the deformation are shown.

**FIGURE 6.**
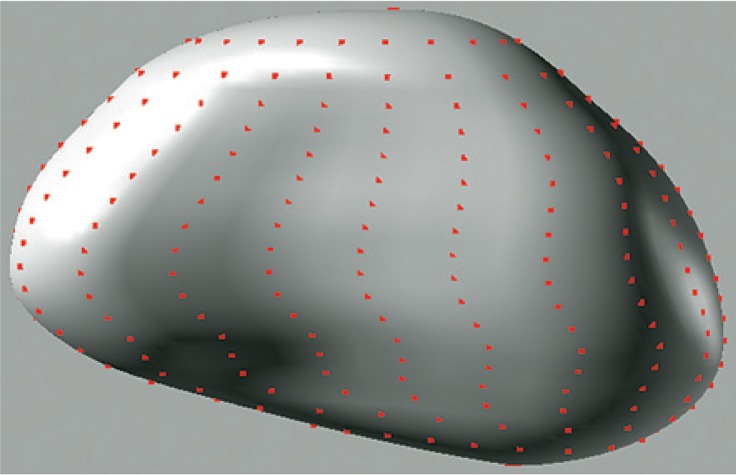
Sampling surface points from non-uniform rational B-spline (NURBS) representation of organ.

Based on the NURBS representation of pelvic organ surface, a set of surface points was re-sampled from the NURBS surface. Figure 6 illustrates the sampled surface points on the NURBS surface of bladder.

**FIGURE 7.**
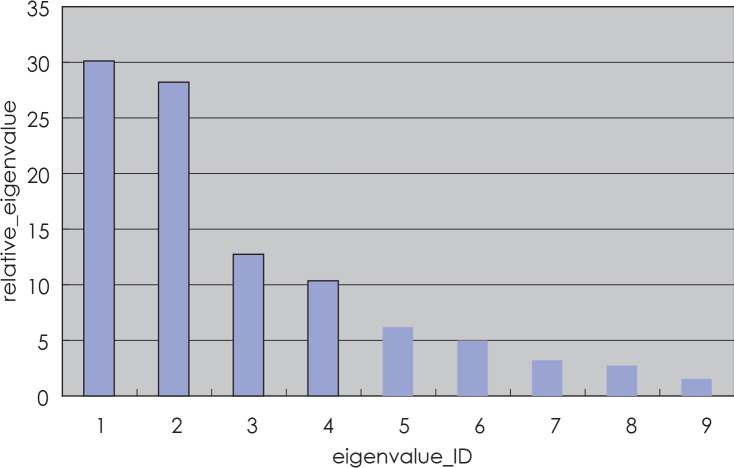
Spectra of relative eigenvalues for training datasets (sum of all eigenvalues normalized to 100%).

PCA was performed on the sampled surface points to capture the major variation modes of the surfaces for the same organ. For the pelvic organs in our study, shape variations have shown to be clearly dominated by only a few eigenvalues, indicating that the geometric variability of the measured organ samples is concentrated in just a few deformation modes. From the statistical shape modeling of pelvic organ, we described the shape variability in the training sets by the first five principal modes, which covered > 90% of the variance in shape change found within the training sets. Figure 7 illustrates the spectra of eigen values of a training dataset.

**FIGURE 8.**
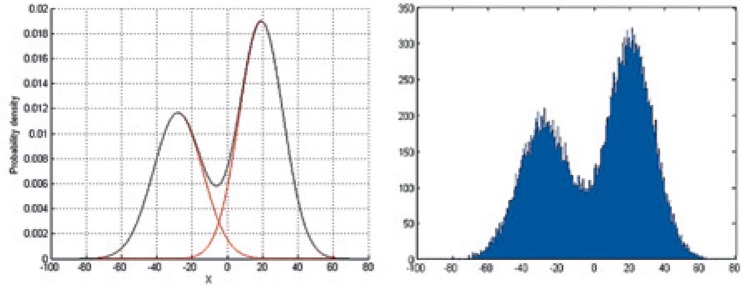
Probability density function (PDF) approximation of coefficient and random coefficients generation. **(A)** PDF of coefficient; **(B)** Random generated coefficient.

Figure 8 illustrates the estimation and the corresponding approximation of the PDF of one of the coefficients *b_q_*, which contains a mixture of two gaussian distributions. Random coefficients were generated to match this kind of mixture-gaussian distribution by Monte-Carlo sampling.

**FIGURE 9.**
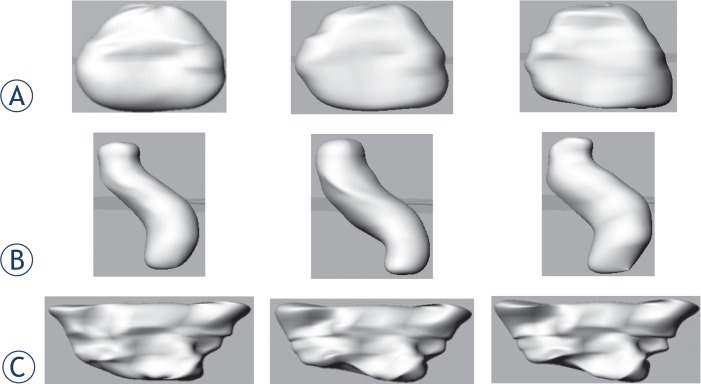
New geometries of organs described by statistical shape models after varying the coefficients corresponding to the principal components. **(A)** Generated new geometries of the bladder; **(B)** Generated new geometries of the rectum; **(C)** Generated new geometries of the intestine.

Based on the statistical shape analysis of training data sets, new geometries of organs have been generated by a combination of eigenvectors and respective coefficients. Examples of new geometries obtained can be seen in Figure 9.

## Discussion

During the course of adaptive radiotherapy, the management of large datasets of organ geometries is a major challenge for clinicians and physicists, not only during image segmentation, but also during the generation of new treatment plans. In this study, we have proposed a novel method to generate large datasets of organ geometries from limited patient data to be used in adaptive radiotherapy. The feasibility of our approach has been illustrated with the construction of new organ shapes from a given limited image set. However, the method itself bypasses the labor of clinical datasets acquisition. Furthermore, the underlying methodology is potentially useful for other medical applications, such as creating a virtual population of anatomical objects for surgical simulation^32^–^33^ or for anatomy education purposes.

The aim of our study has been to derive a realistic model of shape variation via statistical shape modeling, and to obtain new instances of the anatomical representation based on NURBS. This is of particular interest in adaptive radiotherapy, especially due to the increasing demand for on-line organ segmentation, re-planning and re-optimization. Statistical shape modeling can afford an efficient parameterization of the geometric variability of the organ anatomy of patients. The population of variable organs created by our method can be utilized as a data source to support on-line adaptive radiotherapy work. For example, a large number of radiotherapy plans can be generated based on the new geometries. New geometries can also be used to create statistical atlases for segmentation purposes.

Shape representation and shape parameterization are crucial in 3D visualization, image processing, and shape deformation, and determine flexibility, complexity, possible user interactions, and other important issues in various applications.^34^ In our study, NURBS has been used for the organ shape representation. NURBS can offer a unified mathematical formulation for representing not only free-form curves and surfaces, but also standard analytical shapes such as conics, quadrics, and surfaces of revolution.^35^ It has been widely used as a free form transformation technique and it provides additional flexibility to design a large variety of shapes. In addition, it can reduce the storage memory in computerized processing in medical applications.

Accurate segmentation is highly desired in the image analysis, which plays an important role in the whole framework proposed in this paper. 2D contours derived from medical images is the bases of 3D geometry of organs, thus the accuracy of segmentation is correlated with the statistical distribution of organ shapes in some way. Based on an experimental simulation, we found that 1–2 mm segmentation errors in 2D images may result in 3–5 mm displacement in 3D organ shapes, deformation modes and geometrical distribution are also affected with the surface error. With more training shapes, the geometrical distribution of organ variation can be presented with more degrees of freedom, even the related effect of segmentation error may be decreased at some resolution level. The segmentation accuracy also can be improved by introducing multi-modality images and registration, which were often utilized to overcome such challenging problems (*i.e*., low-resolution, blurred boundaries, high noise levels, signal drops, etc.). More realistic representation of organ shape distribution maybe obtained with novel strategy of contour segmentation in the future.

By modifying the position of the control points and separating the deformation into intermediate steps, we have been able to control the surface deformation process. A relatively small deformation step may avoid the local minima problem and guarantee convergence of realistic results. Since B-splines have the feature of local support, iterative warping of control points in the NURBS surface transformation is performed until the desired convergence is reached, which makes the deformed results closer to the target surface.

We have limited our implementation to principal component analysis (PCA), which has shown to be an efficient and effective tool for statistical shape modeling. However, it is possible that some alternative methods might get some improvement in accuracy. The principal geodesic analysis^36^ can also be applied in shape representation and may be more accurate in non-linear shape variation studies. An independent component analysis (ICA) is also used in the shape analysis, especially to capture localized variations. Results of constructing a statistical shape model with ICA and PCA for cardiac MR segmentation have been compared, showing that ICA-based model yielded more accurate segmentations over PCA-based model.^37^

Although showing promising potential, there is still much work to be done and much to improve in the methodology here proposed. If intensity information can be assigned to the generated geometries, CT-like medical images could be generated for dosimetric calculations. Deformable multi-organ registration technique, often required in radiation therapy, allows various organ shapes to behave differently and maintain the geometric integrity of different organs. More work is, therefore, necessary to statistically analyze the correlation and interactions of multi-organs, which can be used to build multi-organ models and aid deformable registration.

## Conclusions

A method for automatic generation of large data-sets of organ geometries from a limited set of images has been developed. The generated organ geometries are realistic and statistically representative of anatomical changes. We have shown that it is possible to capture a major portion of the total shape variability with the first few eigenvectors, and we have validated the creation of new geometric instances based on statistical shape analysis and NURBS representation.

This technique shows potential as a method for application of adaptive radiotherapy in cases with limited patient datasets. Future directions include exploring intensity models and multi-organ models for dosimetric simulation and deformable registration during adaptive radiotherapy.
